# Discontinuation of contraceptives among adolescent girls aged 15–19 years in Nigeria: a descriptive analysis

**DOI:** 10.4314/ahs.v22i4.37

**Published:** 2022-12

**Authors:** Olaide Ojoniyi, Kanayo Ogujiuba, Nancy Stiegler

**Affiliations:** 1 Department of Statistics and Population Studies, University of the Western Cape, South Africa; 2 School of Development Studies, University of Mpumalanga, South Africa

**Keywords:** Contraceptive, discontinuation, adolescents, unmarried, NDHS, Nigeria

## Abstract

**Background:**

Adolescent girls are the mostly affected with maternal and child complications. Contraceptive use is an important tool in curbing sexual and reproductive health challenges especially among adolescent girls in the developing countries. Despite, the low use of contraceptives among adolescents in Nigeria, the possibility of discontinuation of use after initiation is strong.

**Objective:**

This study aims to identify method of contraceptive use discontinued and reasons for discontinuation of contraceptives among unmarried adolescents aged 15–19 years in Nigeria.

**Method:**

Data for 324 never married adolescent girls who had ever used a method to prevent pregnancy were drawn from the 2018 Nigerian Demographic and Health Survey. Descriptive statistics and chart were used to present the results.

**Result:**

Findings showed that 20% of adolescents who had ever used contraceptive discontinued use in the last five years. Most of single adolescents who reported ever discontinued a contraceptive method are older, have secondary education, resides in Urban areas, were at least 15 years at sexual debut are from richer household. Most reported reasons for discontinuation were Infrequent sex and inconveniencey in use.

**Conclusion:**

One in five of unmarried adolescents who ever use a method, discontinue use because of sexual frequency and type of method used.

## Introduction

Discontinuation of contraceptive use when a woman is not ready for pregnancy hampers her reproductive plans, causes unintended pregnancy, and unsafe abortion. Generally, this put women at risk of maternal morbidity and mortality. Seasonal or intermittent use of contraceptives in a growing population has effect on the population structure and has been found attributable to 35% of unwanted pregnancy in developing countries^1^. However, young adolescent girls are more at risk of contraceptive discontinuation than older women and are even more likely to become pregnant while using contraceptive which contributes additional 25 percent to contraceptive failure rate^2^. When single adolescents who experiment with sex discontinue contraceptive, and get pregnant, their education and employment opportunities are deterred, this is not good for countries (e.g., Nigeria) with young people^3^. Nigerian population increases by at least 2.54% every year^4^. Adolescents contribute significantly to new births annually with 23% of adolescent girls aged 15–19 years already entered motherhood 5. Similarly, adolescent girls with unintended pregnancies may resolve to unsafe abortion due to societal factors around premarital pregnancy and legal status of abortion^6^. In Nigeria where access to abortion is legally forbidden, abortions are often performed secretly and are unsafe resulting in serious complications this means that contraceptive discontinuation put Nigerian adolescents at risk of maternal morbidity and mortality^7^.

Contraception has high impact on maternal and child health outcomes with additional benefits for young people such as keeping them in school, employment potential and preventing early motherhood. In spite of the benefits and efforts at all levels globally to encourage contraceptive use, over 200 million women of childbearing age in developing countries including adolescent girls are not ready to get pregnant but are not using contraceptives^8^. It has also been reported that 28 percent of adolescent girls with unintended pregnancy were formerly using contraceptive but discontinued use^9^. Despite the knowledge on the need for contraceptives the rate of discontinuation is on the increase among users who are still at risk of unwanted pregnancy in Nigeria^10^. Comprehending contraceptive discontinuation is important because of its effect on women life course, most importantly for young women with self-motivated contraceptive behaviour 11.

As efforts are been made to ensure adolescent girls sexual and reproductive health rights. It is important to investigate the factors of contraceptive discontinuation that can hinder adolescent girls from meeting their reproductive needs. Several factors have been found to be associated with contraceptive discontinuation among women of reproductive age. Nigerian women of reproductive ages who experience intimate partner violence are more likely to discontinue use of contraceptive^12^. Also, variation in ethnic group and other socio-economic characteristics are associated with contraceptive discontinuation^10^,^13^. Lack of counselling on family planning from a health care provider and who made decision about the method to use also contribute to discontinuation^13^, ^14^.

Although, studies have established association between socio-economic characteristics of women and contraceptive discontinuation among all women. Little is known about unmarried adolescents who discontinues the use of contraceptives in Nigeria.

## Method

Data for this study come from the cross-sectional survey of the 2018 Nigerian Demographic and Health (NDHS) survey. The survey is nationally representative and covers a wide range of topics including contraceptives. The survey used the sample frame from the Nigeria 2006 Population. Census sampling for the survey was stratified in two stages. The states were divided into rural and urban areas and households were selected through probability sampling. Data were collected from women aged 15–49 years for the women recode. In this study, data for 324 single adolescent girls aged 15–19 who had ever used any contraceptive method were used.

The variable of interest is contraceptive discontinuation. It is measured as ‘ever discontinued a method in the last five years. During the survey the respondents were asked if they have ever stopped using a method in the last five years, the method they discontinued and the reasons for discontinuation. The background characteristics used in this study includes age, highest level of education, religion, place of residence, region of residence and household wealth quintile.

Frequency and percentage distribution were used to describe the profile of the study sample, reasons and are presented in tables, pie and bar charts. Socio-economic characteristics of respondents who reported ever discontinued a method in the last five years was assessed. Also, reasons for discontinuation were check against method discontinued. Analysis was weighted to control for sampling error. The statistical package STATA version 14 was used to perform data management and analysis.

Data for this study is extracted from a de-identified open source hence, it is exempted from ethical review by the Ethics committee of the University of the Western Cape, South Africa.

## Result

**Figure 1:**
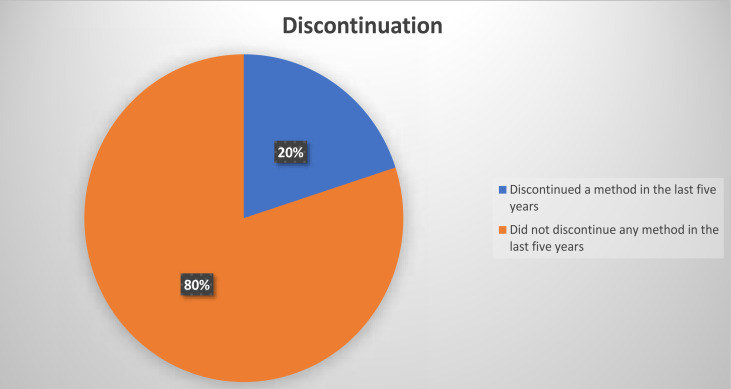
Percentage distribution of discontinuation of any method in the last five years among never-married adolescents in Nigeria.

**Figure 2:**
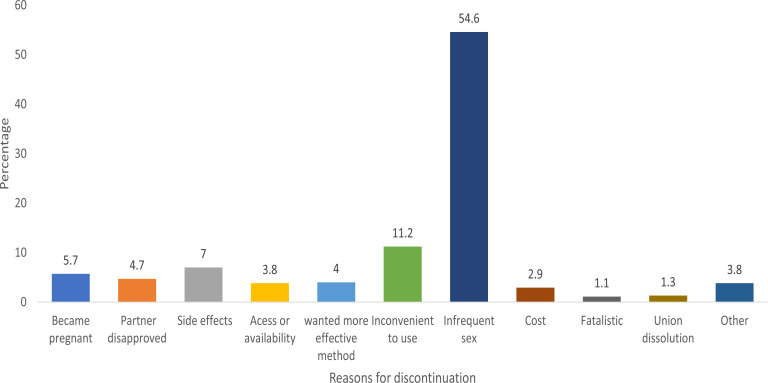
Percentage distribution of reasons for contraceptive discontinuation among unmarried adolescent girls aged 15–19 years in Nigeria.

This section presents the prevalence of contraceptive discontinuation among unmarried adolescents aged 15–19 years within the last five years in Nigeria (figure 1). Table 1 shows the socio-economic characteristics of adolescents aged 15–19 years who discontinued use of any method of contraceptive within the last five years before the survey. Figure 2 depicts the percentage distribution of reported reasons for contraceptive discontinuation among the respondents while table 2 presents the percentage distribution of method discontinuation by reason among unmarried adolescents aged 15–19 years in Nigeria.

**Table 1:** Profile of adolescents aged 15–19 years who discontinued use of any method of contraceptive within the last five years before the survey (NDHS2018)

Variable	Category	Frequency	Percentage
Age	15	1	0.7
	16	7	10.3
	17	13	20.6
	18	21	32.8
	19	23	35.6
	Mean=16.6 Sd=1.4		
Residence	Urban	40	60.5
	Rural	26	39.5
Education	Primary	6	8.6
	Secondary	57	86.5
	Higher	3	4.9
Region	North Central	11	17.0
	North-East	7	10.1
	North-West	2	3.1
	South-East	18	27.8
	South-South	14	20.8
	South-West	14	21.1
Religion	Catholic	14	22.1
	Other Christians	36	55.5
	Islam & Others	15	22.4
Age At First Sex	Below 15 Years Old	12	18.5
	Age 15 Or above	54	81.5
Household Wealth Quintile	Poorest	5	7.3
	Poorer	7	10.8
	Middle	17	26.3
	Richer	21	32.4
	Richest	15	23.2

Note: weighted frequencies and percentages. Percentages may not add up to 100 due to rounding.

**Table 2:** Percentage distribution of method discontinuation by reason among unmarried adolescents aged 15–19 years in Nigeria

Last method discontinued in the last five years	Reason of last discontinuation											
	Became pregnant	Partner disagrees	Side effect	Access unavailable	Wanted more effective method	Inconvenient	Infrequent sex	Cost	Fatalistic	Union dissolution	Other	Total
Pill	0.0	0.0	100	0.0	0.0	0.0	0.0	0.0	0.0	0.0	0.0	1.0
Male condom	2.8	6.9	0.0	5.5	4.3	10.6	63.9	4.3	1.5	0.0	0.0	68.2
Periodic Abstinence	0.0	0.0	0.0	0.0	0.0	0.0	21.2	0.0	0.0	19.6	59.2	6.4
Withdrawal	27.4	0.0	0.0	0.0	7.8	24.6	40.2	0.0	0.0	0.0	0.0	13.7
Other traditional method	0.0	0.0	100.0	0.0	0.0	0.0	0.0	0.0	0.0	0.0	0.0	6.0
Lactational amenorrhea	0.0	0.0	0.0	0.0	0.0	100.0	0	0	0	0.0	0.0	0.6
Emergency contraception	0.0	0.0	0.0	0.0	0.0	0.0	100.0	0.0	0.0	0.0	0.0	2.0
Other modern method	0.0	0.0	0.0	0.0	0.0	0.0	100.0	0.0	0.0	0.0	0.0	2.1
Total	5.7	4.7	7.0	3.8	4.0	11.2	54.5	2.9	1.0	1.2	3.8	100.0

Note: weighted percentages.

For this study, one-fifth (20%) of unmarried adolescent girls who had ever use contraceptive report discontinuation in the last five years.

The background characteristics of the respondents who had ever discontinued a method are shown in table 1. One-third of adolescents who had ever discontinue contraceptive are 19 years. Majority of the respondents who reported discontinuation in the last five years have secondary education while very few has only primary education (8.6%). Most of them live in Urban areas (60.5%). More than one-quarter (27.8%) of adolescents who has ever discontinue use are in the South eastern part of the country while 3.1% lives in the North west. More than half of adolescents who discontinue use within the last five years are Christians apart from Catholic. Majority of adolescents who discontinued use initiate sex at age 15 and above; 32.4% are from the Richer household.

From Figure 2 above, more than half (54.6%) of adolescent who discontinued contraceptive in the last five years did so because of infrequent sex, 11.2% discontinued use as a result of inconvenience in use, this is followed by 7.0% who discontinued use because of side effects. Very few (1.1%) reported fatalistic as the reason for discontinuation.

### Contraceptives among Adolescents

Male condom is the method mostly discontinued in the last five years among adolescents (68.2%), while lactational method is the least method (0.6). Over one-tenth (13.7%) discontinued withdrawal method. While 1.0% discontinued pills.

All who discontinued pill discontinued because of side effects. Majority of those who discontinued male condom did so because of infrequent sex (63.9%) while one-tenth (10.6%) reported inconvenience as reason for discontinuation followed by partner's disagreement.

### Contraceptives among Adolescents

More than half (59.2%) of those who discontinued periodic abstinence did so because of other reasons followed by infrequent sex (21.2%) then union dissolution (19.6%). Two-fifths of those who discontinued withdrawal method did so because of infrequent sex (40.2%) while 27.4% did so because they became pregnant and 24.6% because it is not convenient. All respondents who reported other traditional method as method discontinued in the last five years discontinued the methods because of side effects. Also, all those who stopped emergency contraception and other modern method did so because of infrequent sex. In the same vein those who discontinued LAM discontinued because it is inconvenient for them.

## Discussion

The objective of this study is to examine the reasons why unmarried adolescents aged 15–19 years in Nigeria who had ever use any method to prevent pregnancy has ever discontinued use in the last five years. This study found that one-fifth of adolescents (20%) aged 15–19 years have discontinued a method of contraceptive in the last five years. This is lower compared to the prevalence reported in 2014 for all women of reproductive age in Kenya (30.5%), the prevalence reported among all married women across ethnic groups in Nigeria (Hausa/Fulani 40.2%, Igbo 35.6%, Yoruba 33.6% and Minority 37.8%) 10, and the prevalence reported among all women of reproductive age in Urban Senegal (34.5%), Ethiopia (27.1%), Ghana (56%)^15^–^17^. This may be because of the study population. Most of unmarried adolescents who discontinued method are 19 years old, have secondary education, resides in the Urban areas, had first sex at age 15 or older, from richer household and lives in the Southeast.

The most reported discontinued methods are the male condom, withdrawal, and periodic abstinence while the least reported are the pills, emergency contraceptives, and other modern method (IUD/Implants). This could mean that adolescents contraceptive users prefer male condom more than other type of contraceptives. Male condom is the most reported discontinued method. This is line with findings from a study among undergraduates Brazil which shows that male condom is the most predominantly method discontinued^18^. The study further shows that ladies in spontaneous relationships are at higher risk of male condom discontinuation. This may also explain the situation here as the prevalent reason for male condom discontinuation is infrequent sex. This is also similar to the finding in northern Tanzania among women aged 16–44 years and in Urban Senegal among women of reproductive age, male condom has the highest rate of discontinuation^15^, ^19^.

The most cited reason for discontinuation of all method of contraceptive are infrequent sex, inconveniency, and side effects. These were the same reasons given for discontinuation apart from pregnancy by women of reproductive age in Tanzania^20^. This is similar to the findings among married women of reproductive age in the country, infrequent sex is the most reported reason for discontinuation among married women^10^. It could be that unmarried adolescents in Nigeria engage in sexual relation occasionally and only use contraceptive once-off at the event. In this case, the risk of unintended is worthy of note. In support of this finding, side effect was mentioned as one of the major reasons for discontinuation in Senegal and Ethiopia among all women^15^, ^16^. Also, generally among all married women in Nigeria it is reported as one of the major reasons for discontinuation^10^. This is evident in the method use, it explains why there is low use of hormonal methods among unmarried adolescents in Nigeria.

## Conclusion

Despite low use of contraceptives among unmarried adolescents in Nigeria, a large proportion of those who has ever use any method of contraceptive discontinues use. The reasons reported and profile of affected adolescents indicated a need for better sexual and reproductive health education for adolescents. Also, encouraging the uptake of long term modern contraceptive method among adolescents in Nigeria. It has been shown that short term users ae more likely to discontinue use compared to long term users^21^.
